# Glucose metabolism regulates expression of hair-inductive genes of dermal papilla spheres via histone acetylation

**DOI:** 10.1038/s41598-020-61824-3

**Published:** 2020-03-17

**Authors:** Mina Choi, Yeong Min Choi, Soo-Young Choi, In-Sook An, Seunghee Bae, Sungkwan An, Jin Hyuk Jung

**Affiliations:** 1 Research Institute for Molecular-Targeted Drugs, Department of Cosmetics Engineering, Konkuk University, Seoul, 05029 South Korea; 2 Korea Institute of Dermatological Science, GeneCellPharm Corporation, 375 Munjeong 2(i)-dong, Songpa-gu, Seoul, 05836 South Korea

**Keywords:** Dietary carbohydrates, Cell signalling

## Abstract

Cellular metabolism is one of the crucial factors to regulate epigenetic landscape in various cells including immune cells, embryonic stem cells and hair follicle stem cells. Dermal papilla cells (DP) interact with epithelial stem cells to orchestrate hair formation. Here we show that active DP exhibit robust aerobic glycolysis. We observed decrease of signature genes associated with hair induction by DP in presence of low glucose (2 mM) and glycolysis inhibitors. Moreover, hair shaft elongation was attenuated by glycolysis inhibitors. Interestingly, excessive glucose is able to increase the expression of hair inductive genes and elongation of hair shaft. We also observed glycolysis-mediated histone acetylation is increased and chemical inhibition of acetyltransferase reduces expression of the signature genes associated with hair induction in active DP. These results suggest that glucose metabolism is required for expression of signature genes associated with hair induction. This finding may be beneficial for establishing and maintaining of active DP to generate hair follicle *in vitro*.

## Introduction

The dermal papilla (DP), specialized mesenchymal cells at the base of hair follicle, serve crucial roles in hair follicle formation^1^. DP secrete various factors, which initiate hair follicle formation by activating skin epithelial stem cells^2,3^. Generating hair follicle *in vitro* is a major challenge since DP quickly lose hair-inductive activity during passaging in the conventional two-dimensional (2D) culture^4^. In order to restore intrinsic properties of DP, 3D sphere culture has been investigated as an alternative method. The signature genes associated with hair induction can be partially restored in DP using 3D sphere culture^5^. Compared to 2D, 3D sphere culture offers a more physiological relevant system where cell-cell communication as well as microenvironments is more closely represent *in vivo*. Cells respond to their microenvironment by stimulating various gene transcription and occupy their “niche” to differentiation^6^. Many lines of scientific evidence support that metabolic alteration in 3D culture towards nutrient as well as oxygen diffusion would be crucial for recapitulation of *in vivo*. For example, 3D cultures of human pluripotent stem cell derived cardiomyocytes express high levels of enzymes involved in OXPHOS^7^. And 3D cultures of HepaRG spheroids show robust activation of glucose and lipid metabolism^8^. Similarly, energy metabolism has been shown to be one of the major cues during hair follicle formation. Especially, lactate dehydrogenase (LDHA), an enzyme involved in anaerobic glycolysis is required for hair follicular stem cell activation^9^. Whereas, the relationship between cellular metabolism and functional properties of DP has been poorly understood although DP are one of the primary cells for hair follicle generation^10^. In this study, we determined the relationship between glucose metabolism and intrinsic properties of DP, especially expression of hair inductive genes. Because intrinsic properties of DP are restored in the sDP (sphere cultured DP) but not in the cDP (two dimensional cultured DP) according to the previous report^4^, we employed sDP and cDP to determine glucose metabolism.

## Results

### Glucose metabolism is activated in sDP

**Figure 1:**
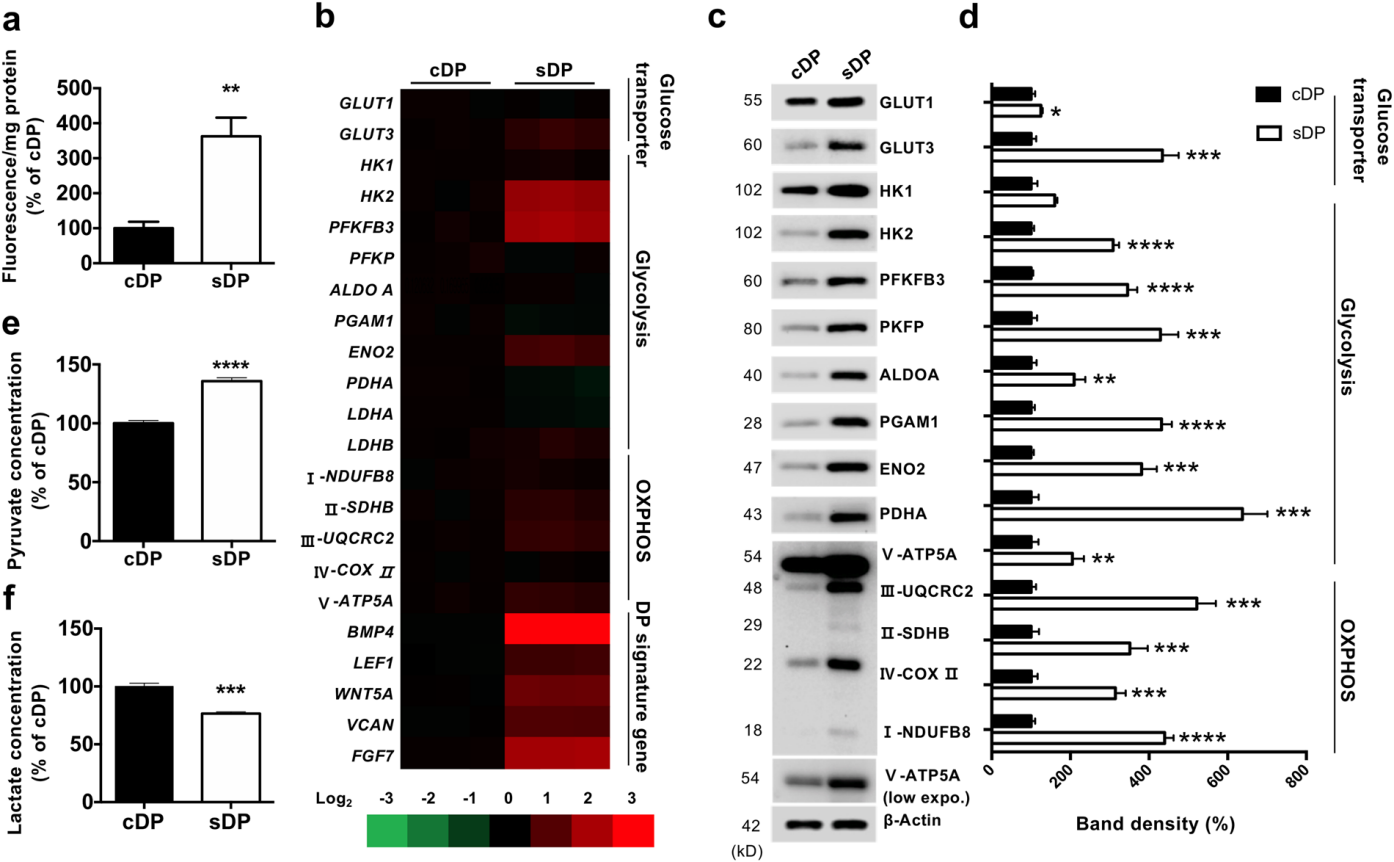
Glucose metabolism is activated in sDP. (**a**) Glucose uptake was measured by transporting of fluorescent Deoxyglucose Analog (2-NBDG) at 80 μM in cultured DP (cDP) and DP spheres (sDP). (**b**) mRNA expression of glucose metabolism and signature gene associated with hair induction was measured in cDP and sDP. (**c**) Protein levels of metabolic enzyme were measured in cDP and sDP. (**d**) Quantification of western blot analysis from three independent experiments (**e**) Intracellular pyruvate was measured in cDP and sDP. (**f)** Extracellular lactate was measured from cDP and sDP. Data (**a**,**d**,**e**) shown are mean ± STE and analyzed by student t-test (*p < 0.05, **p < 0.01, ***p < 0.005, ****p < 0.001).

To measure glucose metabolism, we determined glucose uptake in cDP and sDP using fluorescent 2-NBDG. Glucose uptake was 3-folds higher in sDP compared to cDP, which indicating sDP exhibit robust glycolysis (Fig. 1a). Consequently, the levels of mRNA expression of metabolic enzymes were increased in sDP along with high expression of DP signature genes (Fig. 1b). Protein levels of metabolic enzyme were also increased in sDP (Fig. 1c,d). To determine whether sDP exhibit anaerobic or aerobic glycolysis, we measured pyruvate and lactate levels. sDP showed higher pyruvate levels but not lactate levels, which indicating aerobic glycolysis is activated by sDP (Fig. 1e,f). We also compared expression of the genes related with aerobic glycolysis between 3D cultured DP and paired fresh DP from microarray data (GSE44765_5)^5^. The expression levels of enzymes, which related with glycolysis and oxidative respiration were increased from 3D cultured DP (Table S1). These results indicated that glucose metabolism is induced in sDP along with high expression of hair inductive genes.

### Glucose metabolism is required for the expression of hair inductive genes

**Figure 2:**
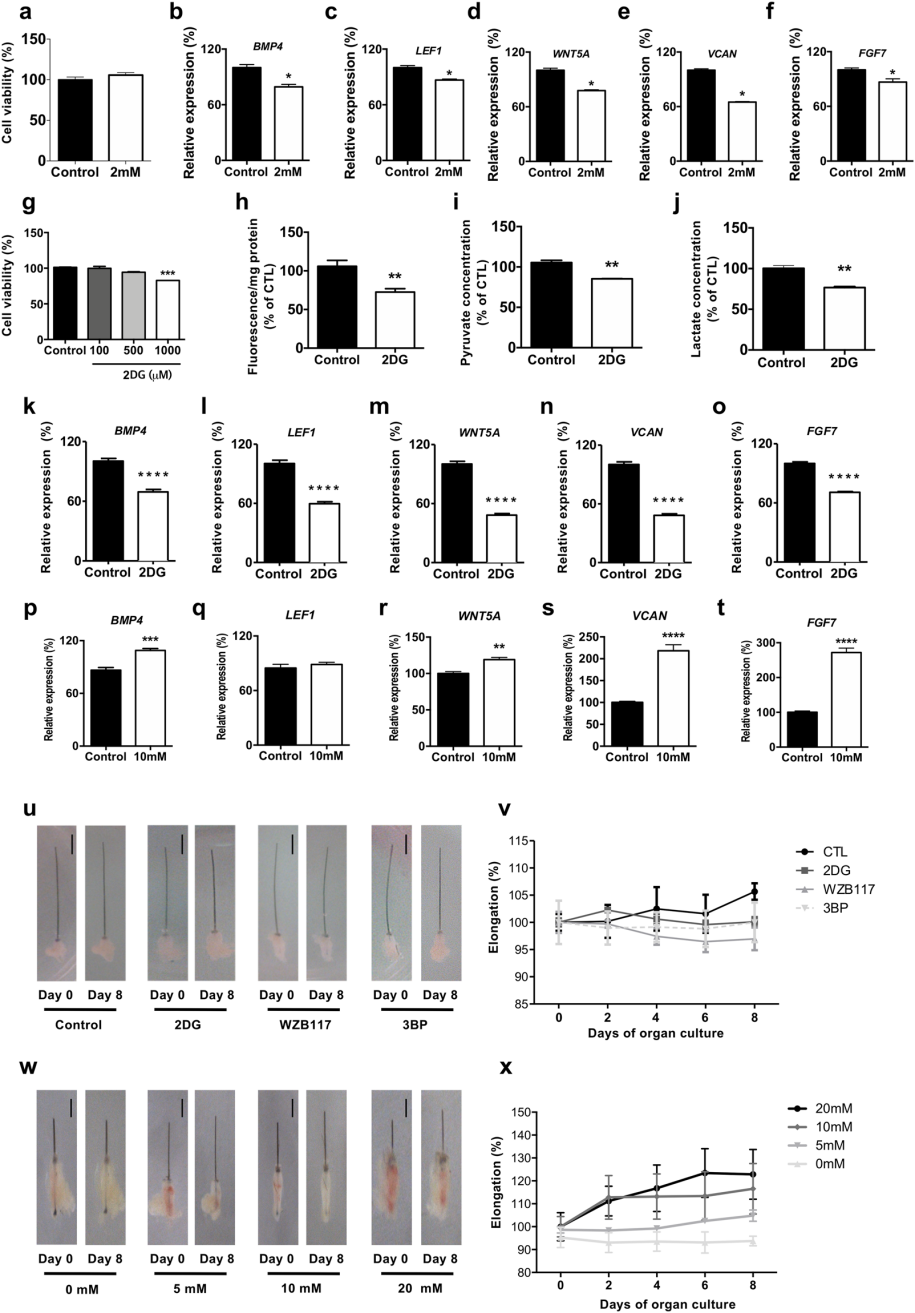
Glucose metabolism is required for the induction of hair inductive genes. (**a**) Cellular viability was measured in sDP after 48 h of incubation with either 5.5 mM (control) or 2 mM glucose incubation. (**b**–**f**) mRNA expression of indicated genes were measured in sDP after 48 h of incubation with either 5.5 mM (control) or 2 mM glucose. (**g**) Cellular viability was measured in sDP presence of indicated concentration of 2 deoxy-d-glucose (2DG) for 48 h. (**h**) Glucose uptake was measured in sDP after 48 h of incubation with either vehicle or 500 μM of 2DG treatment. (**i**) Intracellular pyruvate was measured in sDP after 48 h of incubation with either vehicle or 2DG (500 μM). (**j**) Extracellular lactate was measured from sDP after 48 h of incubation with either vehicle or 2DG (500 μM). (**k**–**o**) mRNA expression of indicated genes were measured in sDP after 48 h of incubation with either vehicle or 2DG (500 μM). (**p**–**t**) mRNA expression of indicated genes were measured in sDP after 48 h of incubation with either control (5.5 mM glucose) or 10 mM glucose treatment in presence of 2DG (500 μM). (**u**) Isolated mice hair follicles were treated with each indicated chemicals of 500 μM of 2DG or 10 μM of WZB 117 or 20 μM of 3-Bromopyruvate (3BP) for 8 days. Images of representative mice hair follicle were taken from each group at day 0 and day 8, and the length of mice follicle was measured. Scale bar = 300 μm (**v**) Quantification of the hair shaft of length over time. (**w**) Isolated mice hair follicles were treated with each indicated glucose concentration for 8 days in presence of 2DG (500 μM). Images of representative mice hair follicle were taken from each group at day 0 and day 8, and the length of mice follicle was measured. Scale bar = 300μm (x) Quantification of the hair shaft length over time. Data (a-t) shown are mean ± STE and analyzed by Student t-test (*p < 0.05, **p < 0.01, ***p < 0.005, ****p < 0.001).

Because sDP exhibit robust glycolysis, we incubated sDP in different glucose concentration to determine whether glucose metabolism is required for the expression of signature genes associated with hair induction. Expression of DP signature genes including *BMP4*, *LEF1*, *WNT5A*, *VCAN* and *FGF7* were decreased from sDP in presence of low glucose (2 mM) compared with physiological of glucose (5.5 mM) without alteration in cellular viability (Fig. 2a–f). Consequently, we used 2-deoxy-d-glucose (2DG) to determine whether inhibition of glycolysis modulates the expression of DP signature genes. 2DG treated DP showed decrease of glucose uptake and glucose-derived metabolites without alteration in cellular viability (Fig. 2g–j). The expression of the signature genes associated with hair induction was decreased by 2DG (Fig. 2k–o). Moreover, expression of the signature genes associated with hair induction was decreased by glycolytic inhibitors including 3BP and WZB117 (Fig. S2a–j). Interestingly, excessive glucose (10 mM) supplement increased expression of the signature genes associated with hair induction (Fig. 2p-t). Consequently, hair shaft elongation was attenuated by glycolytic inhibitors (Fig. 2u,v). We also found that hair shaft elongation was enhanced by increased glucose concentration (Fig. 2w,x). These results suggest that glucose metabolism is required for expression of the genes associated with hair induction in DP.

### Glycolysis-mediated acetylation is required for the expression of hair signature genes

**Figure 3:**
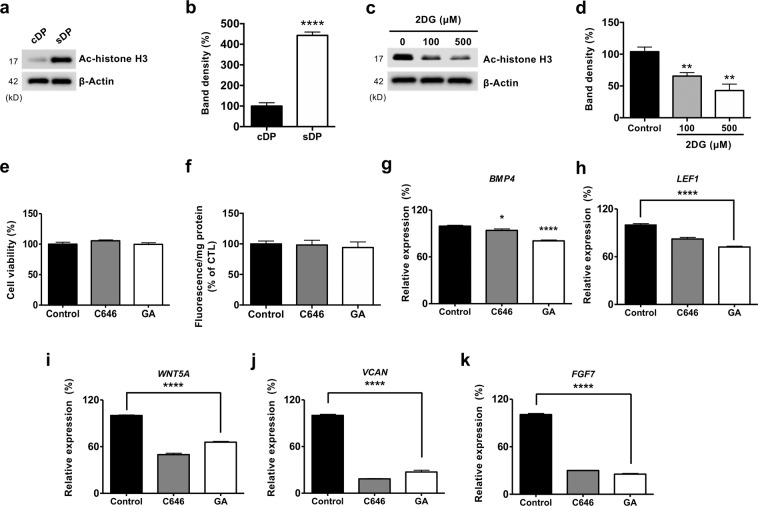
Glycolysis-mediated acetylation is required for the expression of hair signature genes (**a**) Protein levels of acetylated histone were measured in cDP and sDP. (**b**) Quantification of western blots from three independent experiments. (**c**) Protein level of acetylated histone was measured in sDP after 48 h of incubation with indicated concentration of 2DG. (**d**) Quantification of western blots from three independent experiments. (**e**) Cellular viability was measured in sDP after 48 h of incubation with either vehicle or 10 μM C646 or 10 μM Ginkgolic acid. (**f**) Glucose uptake was measured in sDP after 48 h of incubation with either vehicle or 10 μM C646 or 10 μM Ginkgolic acid. (GA). (**g**–**k**) mRNA expression of indicated genes were measured in sDP after 48 h of incubation with either vehicle or 10 μM C646 or 10 μM Ginkgolic acid (GA). Data shown are mean ± STE and analyzed by one-way analysis of variance (ANOVA) (*p < 0.05, **p < 0.01, ****p < 0.001).

Excessive glucose promotes hyperacetylation of histones, which directly activates the expression of genes in nutrient-favorable conditions^11^. To investigate the link between glucose metabolism and the expression of genes associated hair induction in DP, we measured histone acetylation in cDP and sDP. Interestingly, the levels of histone acetylation were increased in sDP (Fig. 3a,b). Moreover, we found high expression levels of histone acetylating enzymes from DP of anagen hair follicle compared with pairing fibroblasts in web-based meta-analysis (GSE31324)^4^ using microarray data (Table S2). Consequently, we measured histone acetylation after 2DG treatment to determine whether glucose metabolism is required for histone acetylation. The levels of histone acetylation were decreased by 2DG treatment (Fig. 3c,d). These results indicated that histone acetylation is regulated by glycolysis in DP. We used two histone acetyltransferase inhibitors, C646 and Ginkgolic acid to determine whether glucose-mediated histone acetylation is required for the expression of genes associated with hair induction in DP. The expression of genes associated with hair induction was decreased by these inhibitors without alteration in cellular viability as well as glucose uptake (Fig. 3e–k). These results suggested that DP rely on glycolysis for the expression of DP signature genes through histone acetylation.

## Discussion

DP showed metabolically less active compared with neighboring cells in plucked hair according to previous report^12^. Our findings suggest that active DP rely on aerobic glycolysis that differs from hair follicle stem cells, which exhibit anaerobic glycolysis^9^. Compartmentation of metabolism in the tissue have been reported including astrocyte-neuron metabolic cooperation^13^. Therefore, it would be interesting to investigate metabolic interplay between DP and hair follicle stem cells during hair cycle as well as hair regeneration. Glycolysis is required for the expression of genes associated with hair induction (Fig. 2). Carbohydrates including galactose, glucosamine and fructose are transported through glucose transporters and utilized in glucose metabolism^14,15^. Therefore, excessive carbohydrates-mediated DP inductivity should be determined in the future. On the other hands, glycolysis-mediated histone acetylation promotes differentiation of embryonic stem cells and proinflammatory cytokine production in helper T cells through epigenetic control^16,17^. Based on these scientific backgrounds, we hypothesized that the incorporated excessive glucose into sDP promotes signature gene transcription by histone acetylation. We observed high levels of histone acetylation in sDP (Fig. 3a,b). Moreover, histone acetylating enzymes including KAT6A and KAT6B were increased in DP from anagen hair follicles (Table S2). Glycolysis inhibitor, 2DG inhibits histone acetylation in sDP (Fig. 3c,d). Our data suggest glucose is required for histone acetylation, which is important for the expression of hair inductive genes (Fig. 3). Interestingly, our data correlates with recent studies that histone deacetylase inhibitors including tricostatin A (TSA) and valproic acid promote hair inductivity^18,19^. Taken together, our finding suggests the standpoint where we could see a novel metaboloepigenetic regulation of DP although fine tuning of histone acetylation and epigenetic regulation of DP signature genes would be remains to explore in the future.

## Methods

All methods were performed in accordance with the relevant guidelines and regulations

### Cell culture and reagents

Dermal papilla cells (DP; PromoCell GmbH, Heidelberg, Germany, Lot 325Z017.1 and 322Z030.1) were maintained in the Follicle Dermal Papilla Cell Basal Medium (PromoCell GmbH) supplemented with 4 µl/ml bovine pituitary extract, 0.04 ml/ml fetal calf serum, 1 ng/ml basic recombinant human fibroblast growth factor, 5 µg/ml recombinant human insulin (PromoCell). Cells were cultured with 50 mg/ml primocin (Invivogen, Toulouse, France, #ant-pm-2) in a humidified 5% CO_2_ incubator at 37 °C. cDP and sDP were maintained as previously reported^4^. Briefly, cDP were maintained in 60 mm dish (SPL, Gyeonggi-do, Korea, #20060) and sDP were maintained in Ultra Low Attachment Culture Dish (Corning, New York, USA #3261). Both cDP and sDP were maintained same DP media described above. 2-deoxy-d-glucose (2DG), WZB117 and Bromopyruvic acid (3BP) were purchased from Sigma–Aldrich (St. Louis, USA, #D8375, #SML0621, #16490 respectively). D-(+)-Glucose powder were purchased from Sigma–Aldrich (#G7021). C646 (#10549) and Ginkgolic acid (#18422) were purchased from Cayman Chemical (Ann Arbor, USA).

### Cell viability assay

The viability of the DP was measured using the water-soluble tetrazolium salt (WST-1) assay (EZ-Cytox Cell Viability Assay Kit; ITS-Bio, Seoul, Korea, # EZ3000) according to previously described^20^. Absorbance was determined at 450 nm using an iMark microplate reader (Bio-Rad laboratories, Hercules, USA).

### 2-deoxy-d-glucose ransport Assay

DP were cultured for 48 h with 80 μM of the fluorescent 2-deoxy-d-glucose analog, 2-NBDG (2-deoxy-2-[(7-nitro-2,1,3- benzoxadiazol-4-yl)amino]-D-glucose, Cayman, #186689-07-6) in glucose free media for 30 min. Reactions were terminated by solubilizing cells according to previously described^14^. Briefly, 2-NBDG fluorescence was measured at 485/538 nm (excitation/emission) and fluorescent values were normalized to the respective protein concentration using BCA protein Assay kit (Thermo Fisher Scientific, Rockford, USA, #23225).

### Quantitative real-time PCR analysis

Total cellular RNA was isolated from DP using TRIzol reagent (Thermo Fisher Scientific, # 15596-018) and cDNA was synthesized using M-MLV reverse transcriptase (Thermo Fisher Scientific, #28025-013) according to previously decribed^21^. Quantitative reverse transcription polymerase chain reaction analysis was performed using SYBR™ green PCR master mix (Thermo Fisher Scientific, #4309155) with a Step OnePlus Real-Time PCR System (Thermo Fisher Scientific). The sequences of the primer sets are listed in Table S3. mRNA levels were normalized to human 18 S rRNA. Data shown are mean ± STE and analyzed by Student t-test (*p < 0.05).

### Western blot analysis

Western blot analysis was performed as previously described^22^. Sources and working dilutions of antibodies are shown in Table S4. Each protein was detected using enhanced chemiluminescence (Bio-Rad laboratories, #1705061) and visualized using the ChemiDoc Touch Imaging System (Bio-Rad laboratories).

### Pyruvate and Lactate assays

Concentrations of intracellular pyruvate and extracellular L-lactate were measured fluorometrically (ex 544 nm, em 590 nm) using kits from Cayman Chemical (#700470, #700510 respectively) according to previously described^14^ and fluorescent values were normalized to total protein for each well determined using BCA protein Assay kit (Thermo Fisher Scientific, #23225).

### Hair shaft elongation assay

Hair shaft elongation assay was performed according to previously described^23–25^. For organ cultures of mouse vibrissa follicles, individual hair follicles were dissected from the upper lip pad of 5-week-old female C57BL/6 mice. Vibrissa follicles were placed individually in 24-well plates containing Follicle Dermal Papilla Cell Basal Medium (PromoCell GmbH) supplemented with 4 µl/ml bovine pituitary extract, 0.04 ml/ml fetal calf serum, 1 ng/ml basic recombinant human fibroblast growth factor, 5 µg/ml recombinant human insulin (PromoCell) with 1 x penicillin- streptomycin solution (Gibco; Invitrogen, Carlsbad, CA, USA, #15140-122) and each chemical was added to the culture medium. Protocols approved by the Institutional Animal Care and Use Committee (Konkuk University, Republic of Korea). No. KU16199

### Web-based meta-analysis

Microarray datasets from studies (GSE44765_5^5^ and GSE22623722^4^) were analyzed using GEO2R (https://www.ncbi.nlm.nih.gov/geo/geo2r) to determine enzyme expression of aerobic glycolysis and histone acetylation.

### Ethics approval and consent to participate

All animals were care for by using protocols approved by the Institutional Animal Care and Use Committee (Konkuk University, Republic of Korea). No. KU16199.

### Statistical analysis

Data were analyzed by two-tailed Student t-test, one-way analysis of variance (ANOVA) using GraphPad Prism software v.5 Unless indicated otherwise, all experiments were performed using triplicate culture dishes and data shown are the mean ± SEM. Two-group comparisons were analyzed by two-sided Student’s t test. p values were calculated, and p < 0.05 was considered significant.

## Supplementary information

Supplementary information.

## Data Availability

All study data are available from the corresponding author upon request.

## References

[1] Driskell RR, Giangreco A, Jensen KB, Mulder KW, Watt FM (2009). Sox2-positive dermal papilla cells specify hair follicle type in mammalian epidermis. Dev..

[2] Rendl M, Polak L, Fuchs E (2008). BMP signaling in dermal papilla cells is required for their hair follicle-inductive properties. Genes. Dev..

[3] Zhang H (2016). iTRAQ-Based Quantitative Proteomic Comparison of Early- and Late-Passage Human Dermal Papilla Cell Secretome in Relation to Inducing Hair Follicle Regeneration. PLoS One.

[4] Ohyama M, Kobayashi T, Sasaki T, Shimizu A, Amagai M (2012). Restoration of the intrinsic properties of human dermal papilla *in vitro*. J. Cell Sci..

[5] Higgins CA, Chen JC, Cerise JE, Jahoda CA, Christiano AM (2013). Microenvironmental reprogramming by three-dimensional culture enables dermal papilla cells to induce de novo human hair-follicle growth. Proc. Natl Acad. Sci. USA.

[6] Simon MC, Keith B (2008). The role of oxygen availability in embryonic development and stem cell function. Nat. Rev. Mol. Cell Biol..

[7] Correia C (2018). 3D aggregate culture improves metabolic maturation of human pluripotent stem cell derived cardiomyocytes. Biotechnol. Bioeng..

[8] Takahashi, Y. *et al*. 3D spheroid cultures improve the metabolic gene expression profiles of HepaRG cells. *Biosci Rep* **35**, 10.1042/BSR20150034 (2015). 10.1042/BSR20150034 PMC4613666 26182370

[9] Flores A (2017). Lactate dehydrogenase activity drives hair follicle stem cell activation. Nat. Cell Biol..

[10] Driskell RR, Clavel C, Rendl M, Watt FM (2011). Hair follicle dermal papilla cells at a glance. J. Cell Sci..

[11] Sebastian C, Mostoslavsky R (2017). The Various Metabolic Sources of Histone Acetylation. Trends Endocrinol. Metab..

[12] Williams R, Philpott MP, Kealey T (1993). Metabolism of freshly isolated human hair follicles capable of hair elongation: a glutaminolytic, aerobic glycolytic tissue. J. Invest. Dermatol..

[13] Belanger M, Allaman I, Magistretti PJ (2011). Brain energy metabolism: focus on astrocyte-neuron metabolic cooperation. Cell Metab..

[14] Jung JH, Iwabuchi K, Yang Z, Loeken MR (2016). Embryonic Stem Cell Proliferation Stimulated By Altered Anabolic Metabolism From Glucose Transporter 2-Transported Glucosamine. Sci. Rep..

[15] Mueckler M, Thorens B (2013). The SLC2 (GLUT) family of membrane transporters. Mol. Asp. Med..

[16] Moussaieff A (2015). Glycolysis-mediated changes in acetyl-CoA and histone acetylation control the early differentiation of embryonic stem cells. Cell Metab..

[17] Peng M (2016). Aerobic glycolysis promotes T helper 1 cell differentiation through an epigenetic mechanism. Sci..

[18] Guo L (2019). TSA restores hair follicle-inductive capacity of skin-derived precursors. Sci. Rep..

[19] Lee SH (2012). Valproic acid induces hair regeneration in murine model and activates alkaline phosphatase activity in human dermal papilla cells. PLoS One.

[20] Jung JH (2010). Triad 1 induces apoptosis by p53 activation. FEBS Lett..

[21] Choi M (2020). E3 ligase RCHY1 negatively regulates HDAC2. Biochem. Biophys. Res. Commun..

[22] Jung JH, Wang XD, Loeken MR (2013). Mouse embryonic stem cells established in physiological-glucose media express the high KM Glut2 glucose transporter expressed by normal embryos. Stem Cell Transl. Med..

[23] Robinson M, Reynolds AJ, Jahoda CA (1997). Hair cycle stage of the mouse vibrissa follicle determines subsequent fiber growth and follicle behavior *in vitro*. J. Invest. Dermatol..

[24] Jindo T, Imai R, Takamori K, Ogawa H (1993). Organ culture of mouse vibrissal hair follicles in serum-free medium. J. Dermatol..

[25] Lee J, Wu W, Kopan R (2008). Murine vibrissae cultured in serum-free medium reinitiate anagen. J. Invest. Dermatol..

