# Supplementary material for: Glucose metabolism regulates expression of hair-inductive genes of dermal papilla spheres via histone acetylation
Source: Sci Rep. 2020 Mar 17;10:4887. doi: 10.1038/s41598-020-61824-3 (PMC7078220; doi:10.1038/s41598-020-61824-3)
Supplement: Supplementary file 1 — Supplementary information. [file 41598_2020_61824_MOESM1_ESM.pdf]

SUPPLEMENTARY INFORMATION

Glucose metabolism regulates expression of hair-inductive genes of dermal papilla spheres via histone acetylation

Mina Choi[1,a], Yeong Min Choi[2,a], Soo-Young Choi[2], In-Sook An[2], Seunghee Bae[1], Sungkwan An[1*] and Jin Hyuk Jung[2*]

[1]Research Institute for Molecular-Targeted Drugs, Department of Cosmetics Engineering, Konkuk University, Seoul 05029, South Korea. [2]Korea Institute of Dermatological Science, GeneCellPharm Corporation, 375 Munjeong 2(i)-dong, Songpa-gu Seoul 05836, South Korea

*Corresponding author

[a]These authors contribute equally

Corresponding author contact information

Jin Hyuk Jung Ph.D. Korea Institute of Dermatological Science, GeneCellPharm Corporation, 375 Munjeong 2(i)-dong, Songpa-gu Seoul 05836, South Korea

+82-2-70-7797-2927 (Tel); +82-2-3437-8360 (Fax); Jungjh@skinreseach.or.kr (e-mail)

Sungkwan An Ph.D. Research Institute for Molecular-Targeted Drugs, Department of Cosmetics Engineering, Konkuk University, Seoul 05029, South Korea.

+82-2-450-4054 (Tel); +82-2-3437-8360 (Fax); Ansungkwan@konkuk.ac.kr (e-mail)

List of Supplementary Materials

Supplementary Table 1, related to Figure 1

Supplementary Figure 1, related to Figure 1

Supplementary Figure 2, related to Figure 2

Supplementary Table 2, related to Figure 3

Supplementary Figure 3, related to Figure 3

Supplementary Table 3, related to Methods

Supplementary Table 4, related to Methods

Supplementary Methods, related to Methods.

Table S1. Meta-analysis of enzyme expression associated glycolysis and oxidative respiration from 3D cultured cells vs. paired fresh tissue (GSE44765)

Table 1. Meta-analysis of enzyme expression associated glycolysis and oxidative respiration from 3D cultured cells vs. paired fresh tissue (GSE44765)

	Dermal papilla from occipital scalps - 3D cultured cells vs paired fresh tissue (GSE44765)						
	Gene	Imported Id	Rank	Fold Change	P-Value	Test Expression	Control Expression
Glycolysis	SLC2A1	201250_s_at	1509	**4.7**	0.0233	5743.1	1222.5
	SLC2A3	202497_x_at	n.s.	**n.s.**	n.s.	n.s.	n.s.
	HK1	200697_at	n.s.	**n.s.**	n.s.	n.s.	n.s.
	HK2	202934_at	758	**7.58**	0.0117	4624.6	610
	PFKFB3	202464_s_at	1589	**4.51**	0.0042	50048.5	11104.6
	PFKP	201037_at	n.s.	**n.s.**	n.s.	n.s.	n.s.
	ALDOA	200966_x_at	4808	**2.18**	0.043	87979.3	40313.9
	PGAM1	200886_s_at	5545	**1.99**	0.0127	137315.7	68960.3
	ENO1	201231_s_at	3599	**2.63**	0.0453	58029.5	22092.5
	PDHA1	200979_at	5415	**2.02**	0.0138	3067.2	1520.5
	LDHA	200650_s_at	5460	**2.01**	0.0121	193178.7	96322.7
	LDHB	201030_x_at	4688	**2.22**	0.0007	107167.1	48367.5
		213564_x_at	7561	**1.65**	0.0004	126094.1	76256.7
OXPHOS	NDUFB8	201227_s_at	n.s.	**n.s.**	n.s.	n.s.	n.s.
	SDHB	202675_at	4993	**2.13**	0.0211	5245.1	2466
	UQCRC2	200883_at	1218	**5.49**	0.0291	13789.2	2512.8
	Cox2	n.d	n.d	n.d	n.d	n.d	n.d
	ATP5A1	213738_s_at	10894	**1.31**	0.0009	120779.9	91928.3

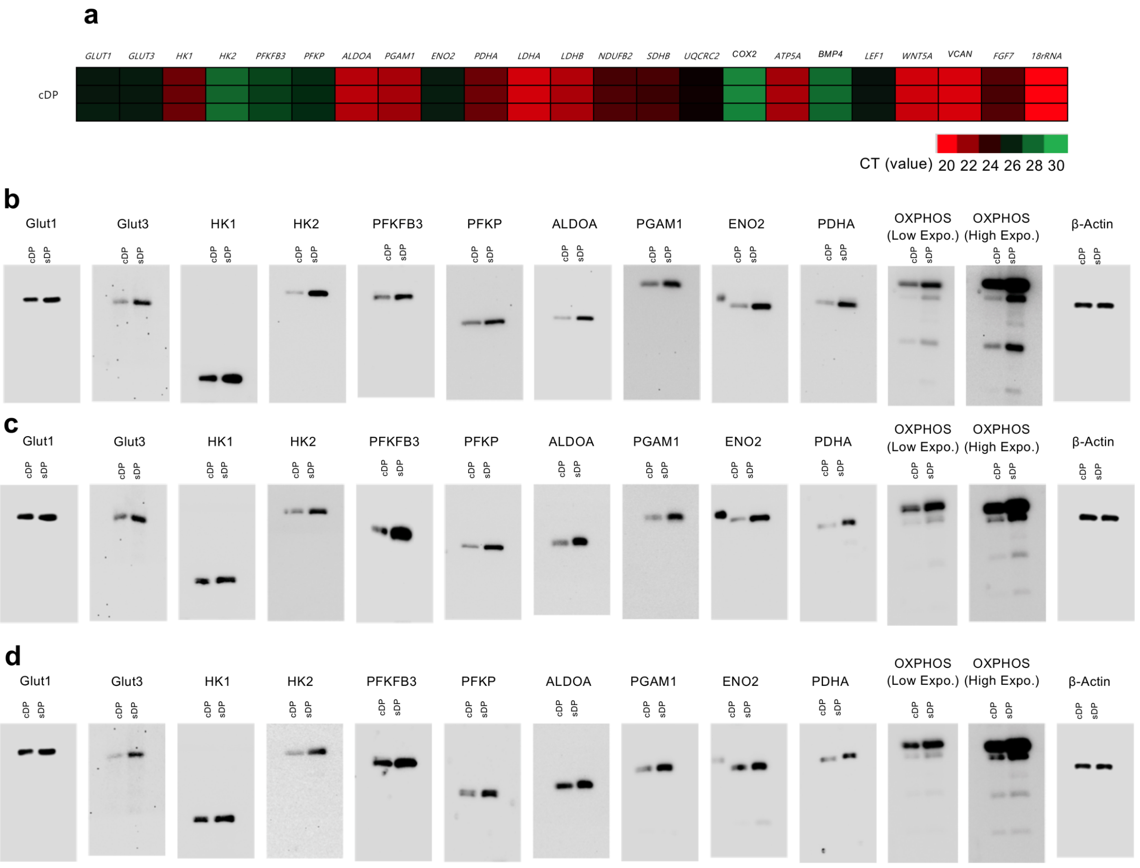

Fig. S1. (a) cDP mRNA expression as form of heat map (CT value) (b)-(d) Full gel images of Fig. 1c

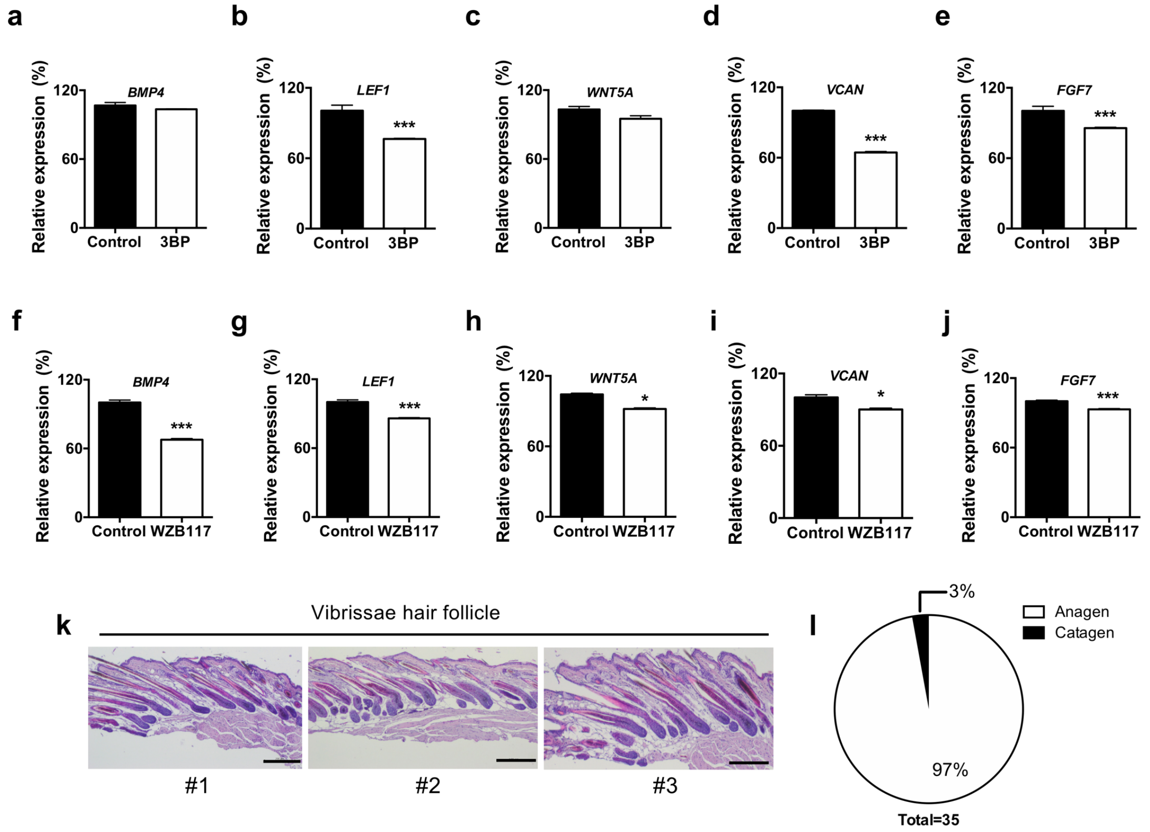

Fig. S2. Glycolysis inhibitors attenuate levels of expression of gene associated with hair induction in sDP. (a-e) mRNA expression of indicated genes were measured in sDP after 48 h of vehicle or 20 μM of 3BP treatment. (f-j) mRNA expression of indicated genes were measured in sDP after 48 h of vehicle or 10 μM of WZB 117 treatment. (k) Representative images of H&E staining of vibrissae hair follicle from three 5-week-old mice (#1, #2 and #3). Scale bar= 200μm (i) Total counts of anagen and catagen of vibrissae hair follicle from 5-week-old mice.

Table S2. Meta-analysis of enzyme expression associated histone acetylation from dermal papilla of anagen hair follicle (GSE31324)

Series	Gene	Imported Id	Rank	Fold Change	P-Value	Test Expression	Control Expression	Samples	PMID
GSE31324	KAT6A	202423_at	2847	1.75	0.0048	2495.9	1427.9	Aggregated dermal papilla of anagen hair follicles _vs_ cultured dermal fibroblasts	22623722
	KAT6B	214496_x_at	590	1.93	0.0304	987.8	511.5		
		212452_x_at	613	1.91	0.0063	1074.3	561.9		
		211874_s_at	658	1.83	0.0107	868.1	474		
		211874_s_at	638	1.69	0.0464	869.1	515.4		
	EID1	208670_s_at	699	1.78	0.0064	9531.4	5357.5		
	EID1	208670_s_at	569	1.78	0.0121	9543.1	5347.1	Dermal papilla of anagen hair follicles - aggregated _vs_ cultured	
	KAT2B	203845_at	737	1.6	0.0255	1481.6	925.3		
	KAT6A	202423_at	444	1.41	0.0338	2373.9	1689.4		
	KAT6A	202423_at	2847	1.75	0.0048	2495.9	1427.9	Dermal papilla of anagen hair follicles - aggregated _vs_ parental culture	
	EID1	208670_s_at	191	1.86	0.036	9364.6	5047.3		

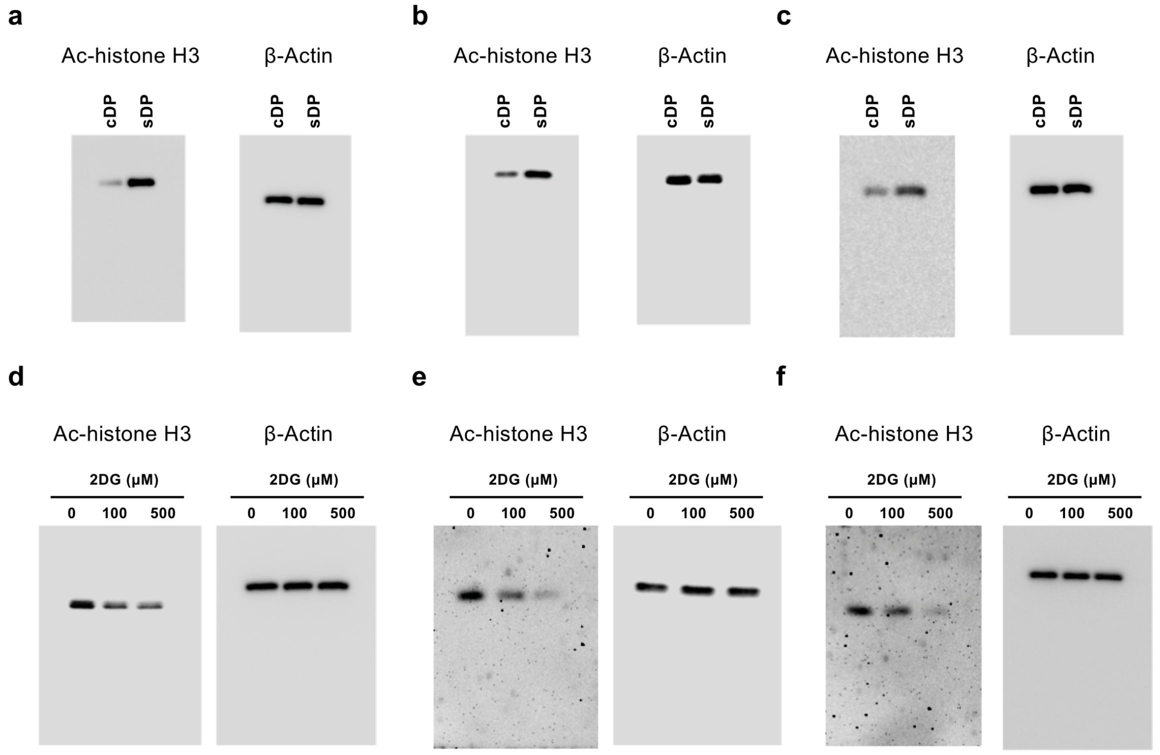

Fig. S3. (a-c) Full gel images from three independent experiments related with Fig. 3a and b. (d-f) Full gel images from three independent experiments related with Fig. 3c and d.

Table S3. List of primers used for Quantitative real-time PCR

Gene	Forward (5' to 3')	Reverse primer (5' to 3')
GLUT1	TGGCATCAACGCTGTCTTCT	AACAGCGACACGACAGTGAA
GLUT3	GACCCAGAGATGCTGTAATGGT	GGGGTGACCTTCTGTGTCCC
HK1	GGACTGGACCGTCTGAATGT	ACAGTTCCTTCACCGTCTGG
HK2	ACTTCCTCGAGTACATGGGC	GAGGAGGATGCTCTCGTCCAG
PFKFB3	CCGTGTGGAATCCATCTACC	CTGTTGATGCGAGGCTTTTT
PFKP	CGGAAGTTCCTGGAGCACCTCTC	AAGTACACCTTGGCCCCCACGTA
ALDOA	GACACTCTACCAGAAGGCGGAT	GGTGGTAGTCTCGCCATTTGTC
PGAM1	ACGTGTACTGATTGCAGCC	CGCCGGCCTTCACTTCT
ENO2	CTGTATCGCCACATTGCTCAGC	AGCTTGTTGCCAGCATGAGAGC
PDHA	AAGAGAGGCGATTTCATTCCTG	TTCACCATCCTGTCCTTGAGAA
LDHA	GGT TGG TGC TGT TGG CAT GG	TGC CCC AGC CGT GAT AAT GA
LDHB	CTG GGA AAG TCT CTG GCT GAT G	CAC TCC ACA CAG CCA CAC TTG A
NDUFB8	AAG CTC CCT GAC CGC TC	CAC GGT TCC TGT TGT ACA TGT C
SDHB	CCC GTA TCA AGA AAT TTG CCA	GGT GAA AGT AGA GTC AAC TTC A
UQCRC2	AAT CCG CAG ACT CAT GTC ATT G	GAC TCA CAC CAA GTC CAA TCA A
COX II	TAC GGC GGA CTA ATC TTC AA	AAA ACA GAT GCA ATT CCC GG
ATP5A	TGC TAT TGG TCA AAA GAG ATC C	CGT AGC CGA CAC CAC AAT
BMP4	GCCCGCAGCCTAGCAA	CGGTAAAGATCCCGCATGTAG
LEF1	CCCGATGACGGAAAGCAT	TCGAGTAGGAGGGTCCCTTGT

WNT5A	TCCACCTTCCTCTTCACACTGA	CGTGGCCAGCATCACATC
VCAN	GGCAATCTATTTACCAGGACCTGAT	TGGCACACAGGTGCATACGT
FGF7	ATCAGGACAGTGGCAGTTGGA	AACATTTCCCCTCCGTTGTGT
18S rRNA	CGGCTACCACATCCAAGGAA	GCTGGAATTACCGCGGCT

Table S4. List of antibodies

Antibody	Dilution	Species	Source
Anti-GLUT1	1:1000	Mouse	Abcam (ab40084)
Anti-GLUT3	1:1000	Mouse	Santa Cruz Biotechnology (sc-74399)
Anti-HK1	1:1000	Rabbit	Cell Signaling Technology (2024)
Anti-HK2	1:1000	Rabbit	Cell Signaling Technology (2867)
Anti-PFKFB3	1:1000	Rabbit	Cell Signaling Technology (13123)
Anti-PKFP	1:1000	Rabbit	Cell Signaling Technology (8164)
Anti-ALDOA	1:1000	Rabbit	Cell Signaling Technology (8060)
Anti-PGAM1	1:1000	Rabbit	Cell Signaling Technology (12098)
Anti-ENO2	1:1000	Rabbit	Cell Signaling Technology (8171)
Anti-PDHA	1:1000	Rabbit	Cell Signaling Technology (3205)
Anti-OXPHOS cocktail	1:500	Mouse	Abcam (ab110411)
Anti-Acetyl histone H3	1:1000	Rabbit	Sigma-Aldrich (06-599)
Anti-Mouse IgG (HRP coupled)	1:2000	horse	Cell Signaling Technology (7076)
Anti-Rabbit IgG (HRP coupled)	1:2000	Goat	Cell Signaling Technology (7074)
Anti-β-Actin	1:12000	Mouse	Sigma-Aldrich (A5441)

Supplementary methods

Histology

The tissues containing vibrissae hair follicles were collected and fixed in 4% formaldehyde solution. Tissues were processed using standard methods (from 70% to 100% ethanol and xylene step) and were embedded in paraffin. Tissues were sectioned into 4μm and then stained by H&E. The stained tissues were observed at 10X magnification under a light microscope (JNOPTIC, AcquCAM 23GR, Seoul, Korea). Pictures were taken using an image acquisition system (JNOPTIC Capture 2.4, Seoul, Korea). Image analysis was calculated as the average of selected three random fields per each mouse. To observe morphology, H&E staining was performed previously describe with slight modification[1]. Tissue slides were de-paraffinized using xylene and hydrated using ethanol in decreasing concentrations (100%, 90%, 80%, and 70%), stained with Harris hematoxylin (Youngdong diagnostics, Youngin, Korea) and Eosin (Sigma-Aldrich, Kenilworth, IL, USA). Next, tissue slides were dehydrated by reverse step of ethanol and xylene, and mounted by Eukitt® Quick-hardening mounting medium (Sigma, USA). Hair cycle was measured according to previously described[2,3]

1 Jung, J. H., Wang, X. D. & Loeken, M. R. Mouse embryonic stem cells established in physiological-glucose media express the high KM Glut2 glucose transporter expressed by normal embryos. *Stem Cells Transl Med* **2**, 929-934, doi:10.5966/sctm.2013-0093 (2013).

2 Ishimatsu-Tsuji, Y., Moro, O. & Kishimoto, J. Expression profiling and cellular localization of genes associated with the hair cycle induced by wax depilation. *J Invest Dermatol* **125**, 410-420, doi:10.1111/j.0022-202X.2005.23825.x (2005).

3 Teta, M. *et al.* Inducible deletion of epidermal Dicer and Drosha reveals multiple functions for miRNAs in postnatal skin. *Development* **139**, 1405-1416, doi:10.1242/dev.070920 (2012).
